# Water–Sediment Partitioning of a Neutrophilic Iron-Cycling Microbiome in the High-Altitude Dzhily-Su Mineral Springs (Mt. Elbrus)

**DOI:** 10.3390/ijms27198549

**Published:** 2026-09-24

**Authors:** Anna Olegovna Izotova, Anastasia Victorovna Komova, Lilia Zuberovna Zhinzhakova, Komila Ravshanovna Khazieva, Gennady Sozyrovich Kachmazov, Christina Vitalievna Chaika, Stepan Vladimirovich Toshchakov

**Affiliations:** 1 National Research Center “Kurchatov Institute”, Ac. Kurchatov Square, 1, Moscow 123098, Russia; anu.izotova@mail.ru (A.O.I.);; 2 High-Mountain Geophysical Institute, Lenina Ave., 2, Nalchik 360030, Russia; 3 Faculty of Chemistry, Biology and Biotechnology, North Ossetian State University Named After Kosta Levanovich Khetagurov, Vatutina Str., 44–46, Vladikavkaz 362025, Russia

**Keywords:** Dzhily-Su mineral springs, neutrophilic iron cycling, *Gallionellaceae*, water–sediment partitioning, Nanoarchaeota, 16S rRNA amplicon sequencing

## Abstract

Mineral springs of the Northern Caucasus remain largely uncharacterized by culture-independent approaches. At the high-altitude Dzhily-Su spring complex, where isotope data indicate deep-seated magmatic fluid inputs, planktonic and sediment-associated communities were analyzed using 16S rRNA gene profiling to determine whether these inputs shape the local microbiome. Rather than a distinct magmatically influenced community, the results revealed a pronounced water–sediment partitioning of taxa with known iron-cycling representatives, microaerophilic *Gallionellaceae* dominating the water fractions and facultatively anaerobic *Aeromonas* and *Shewanella* dominating the sediments. Furthermore, analysis of the fine-size (0.22 µm) water fraction highlighted a notable enrichment of candidate lineages and archaea, including a prominent Nanoarchaeota population (58.6% of archaeal reads) lacking identified mesophilic hosts. The predominance of a typical neutrophilic iron-cycling assemblage, together with the observed water–sediment partitioning, is consistent with an important role of local physicochemical gradients in structuring the microbial community despite the unusual geochemical setting of the parent aquifer.

## 1. Introduction

At oxic-anoxic interfaces in groundwater, springs, and seeps, circumneutral iron cycling relies on a tight interplay between microaerophilic Fe(II)-oxidizing (FeOB) and dissimilatory Fe(III)-reducing bacteria (FeRB) [1,2]. Although analogous systems across Europe, Japan, and North America have been studied extensively, the ferruginous carbonate springs of the Greater Caucasus have so far escaped detailed microbiological characterization. On the northern slope of Mt. Elbrus (Kabardino-Balkarian Republic, Russia), a cluster of cold-to-warm CO_2_-rich mineral springs discharges along a regional geothermal anomaly extending 60–80 km around the edifice. Elbrus is a Late Pleistocene-Holocene stratovolcano with documented magmatic activity within the last two thousand years [3]. Isotopic signatures indicate that these springs represent a mixture of two distinct endmembers: deep-seated magmatic fluids carrying mantle–metamorphic CO_2_ (backed by heavy δ^13^C-CO_2_ values and high ^3^He/^4^He ratios) and meteoric groundwater circulating along fault lines at base temperatures of 190–200 °C [3]. Driven by steep hydraulic head and strong geothermal heating within the Pliocene granite-gneiss basement (Eldzhurtinsky massif), these upwelling fluids mobilize dissolved iron, silica and bicarbonate. Upon discharge, iron-rich waters form extensive ochreous precipitates, a hallmark of Central Caucasus mineral springs that sets them apart from typical calcite-dominated alpine carbonate systems [4].

Located at 2350–2380 m a.s.l. on the northern foot of Mt. Elbrus, the Dzhily-Su spring group on the Malka River has a century-long history of hydrogeological and balneological study [5]. Hydrochemical surveys consistently document stable properties across the primary outlets: slightly acidic to circumneutral pH (5.4–6.7), stable water temperatures of 17–23 °C, total dissolved solids of 3.0–3.6 g L^−1^, a HCO_3_–Ca–Na composition enriched in Cl^−^, silica, boron, and trace elements, and abundant free CO_2_ [3,5,6]. High dissolved iron concentrations qualify these discharges as ferruginous, driving the deposition of characteristic reddish-brown precipitates along the channels. Despite extensive geochemical characterization, the microbial communities at Dzhily-Su remain completely unexamined.

Similar circumneutral, Fe(II)-rich groundwater discharges offer comparative baseline models for microaerophilic iron cycling. At the Fuschna carbonated spring in the Swiss Alps (1260 m a.s.l., pH 6.0–7.0, Fe 12–16 µM, stalk-forming *Gallionella* dominates the microaerophilic Fe-oxidizing community [7]. In a circumneutral iron-carbonate seep in Bloomington (USA), the microbial community exhibits spatial partitioning between a *Gallionellaceae*-dominated planktonic zone (*Gallionella*, *Sideroxydans*, *Aquabacterium*) and a sediment-associated FeRB assemblage (*Rhodoferax*, *Aeromonas*, *Geobacter*), where facultative FeRB couple carbon oxidation to Fe(III) reduction on centimeter scales [8,9]. Investigations of Maine iron seeps [10] demonstrated that low dissolved organic carbon (DOC) and sharp redox boundaries favor exclusive *Gallionellales* assemblages, while higher DOC and diffuse gradients foster *Leptothrix* co-occurrence. Similar trends across Himalayan springs [11], Alaskan tundra pools [12], and coalfield aquifers in South Wales [13] further confirm that local microenvironments, rather than overarching bulk hydrochemistry, govern genus-level community structure. Yet, whether these selection mechanisms operate similarly in high-altitude, magmatically influenced volcanic systems remains an open question.

Here, we present the first 16S rRNA gene amplicon survey of microbial communities across five outlets of the Dzhily-Su spring complex (sampled in August 2022), comparing planktonic (water-fraction) and sediment-associated assemblages. Integrating these molecular profiling data with in situ hydrochemistry and regional isotope-geochemical context, we address three key questions: (i) whether a canonical neutrophilic Fe-cycling consortium persists despite high altitude, magmatic CO_2_ input, and elevated conductivity; (ii) how community structure partitions between water and sediment along a steep redox gradient (Eh from −35 to +75 mV, dissolved O_2_ up to 38% saturation); and (iii) which additional taxa, including candidate lineages and archaeal groups rarely reported from mesophilic carbonate springs expand the known biodiversity and metabolic potential of this system.

## 2. Results

### 2.1. Description of Dzhily-Su Springs

The Dzhily-Su mineral spring complex (“zhyly suu” in Karachay-Balkar, meaning “warm water”) is located on the northern slope of Mt. Elbrus, in the upper reaches of the Malka River, a left tributary of the Terek, in the Republic of Kabardino-Balkaria, Russia. The springs occur at elevations of 2350–2380 m above sea level [3,6], approximately 6 km from the summit of Elbrus and ~80 km from Kislovodsk. The five sampled outlets are distributed within a few hundred meters of one another (43.433–43.434° N; 42.534–42.536° E). Their close spatial proximity is consistent with their location within a single discharge zone of a fracture-hosted carbonate aquifer [3].

#### 2.1.1. Geological and Hydrogeological Setting

The springs are located within Lower Paleozoic metamorphic and igneous basement rocks of the Main Caucasus Ridge, which include granites, gneisses, and amphibolites. These rocks provide a silicate-rich matrix for prolonged water–rock interaction [4]. The Pliocene Eldzhurtinskiy granite massif, associated with post-magmatic activity of the Elbrus Volcanic Centre, is located 3–5 km east of the spring complex. Elbrus is the youngest stratovolcano in the Greater Caucasus, and its most recent documented eruption occurred approximately 2000 years ago. A partially molten magmatic body located at a depth of approximately 2 km generates a geothermal gradient of 130 to 150 °C/km. This geothermal system drives the ascent of mineralized, CO_2_-saturated deep fluids toward the surface [3]. Significant mantle-derived helium components have also been detected in spring gases [14,15], supporting a direct connection between the deep volcanic system and surface discharge. These conditions may support prokaryotic lineages associated with high-temperature or chemolithotrophic niches. Geothermometric estimates indicate reservoir temperatures of 180–250 °C and a fluid circulation depth of approximately 1.5 km. These values contrast with the measured spring discharge temperatures of ~22 °C. Lavrushin attributed this difference to the dilution of deep reservoir fluids by cold meteoric water during their ascent to the surface [3].

#### 2.1.2. Hydrochemistry

The springs discharge weakly mineralized, CO_2_-rich waters, with TDS ranging from 0.7 to 4.0 g/L across the sampled outlets [3,5,6]. The CO_2_ is of mixed volcanic–metamorphic origin and drives active travertine deposition at all discharge points as iron-rich water contacts the atmosphere [3,4]. Published iron concentrations for the main Malka springs reach 6.8–20 mg/L depending on season and year of sampling [4,5]. Iron speciation was not determined during the present sampling campaign. Negative Eh values were recorded at three of the five outlets (−34.9 to −23.2 mV), whereas the upper ‘joints’ outlet showed a value close to zero (+8.0 mV) and the ‘eye’ outlet was more oxidizing (+74.8 mV). Together with the previously reported high total iron concentrations, these physicochemical conditions are compatible with the occurrence of reduced iron at several outlets; however, the relative proportions of Fe(II) and Fe(III) cannot be determined from the available data. The measured pH and oxygen saturation nevertheless fall within the environmental range commonly associated with neutrophilic microaerophilic iron-oxidizing bacteria (Table 1) [7].

#### 2.1.3. Sampling

Samples were collected from five spring outlets in August 2022 (Figure 1). These springs are informally named after the ailments that local residents traditionally associate with their therapeutic use: ‘liver’, ‘stomach’, ‘eye’ and ‘joints’. Although this folk classification has no scientific basis, it provides a practical means of distinguishing the closely spaced outlets and is therefore used throughout the manuscript.

Temperature, pH, redox potential (Eh), conductivity, and dissolved oxygen saturation were measured on-site using a YSI ProDSS Multiparameter Digital Water Quality Meter (YSI Incorporated, Yellow Springs, OH, USA). Coordinates were recorded using a GPS (see Table 1). Total dissolved solids (TDS) were estimated from specific conductance (SpC, µS/cm) using a conversion factor of 0.64 (TDS mg/L = SpC × 0.64), which is consistent with the default freshwater TDS computation applied by YSI instruments.

For the analysis of the microbial community, 5–10 L of water per outlet were sequentially filtered through 0.45 and 0.22 µm Sterivex™ filters (Merck KGaA, Darmstadt, Germany) to retain the particle-associated and free-living fractions, respectively. Sediment samples were collected with sterile spatulas in individual tubes. All samples were fixed with RNAlater immediately after collection and stored at 4 °C during transport.

### 2.2. Analysis of Microbial Diversity Using 16S rRNA Profiling

#### 2.2.1. Sequencing Output and Read Processing

Sequencing of 18 technical replicates across 9 samples yielded a total of 625,403 paired-end reads after adapter trimming. Two replicates of sample 0516A_0.22 were excluded prior to further analysis due to insufficient sequencing depth with only 4653 reads obtained across both replicates, precluding reliable ASV inference. The remaining 16 replicates (8 samples) were processed through the DADA2 pipeline. Quality filtering, denoising, paired-end merging, and chimera removal retained 535,703 reads (86.3% of input). Technical replicates were then merged by sample, producing a final dataset of 8 samples. Read counts ranged from 50,099 reads in 0515A_0.22 to 86,636 reads in 0516E_0.45, with a median of 70,852 reads per sample. Supplementary Table S1 provides the read counts for each replicate at every processing step.

#### 2.2.2. Primary Analysis of Taxonomic Composition and Removal of Human-Associated Allochtonous Taxa

One sediment sample from the ‘joints’ outlet (sample 0516E_sed) contained notable proportions of taxa associated with the human gut microbiome, predominantly *Klebsiella* (42.3%), *Escherichia-Shigella* (13.9%), and *Ewingella* (6.9%). In all other sediment and water-column samples, these taxa occurred only at trace levels (<2%), reflecting localized recreational contamination at site ‘joints’ outlet. Following the filtering criteria described in Section 2.4, these allochthonous reads were excluded from downstream microbial community analyses. The proportion of removed reads was 65.6% in sample 0516E_sed, compared with 0.1–1.9% in all remaining samples (Table 2).

### 2.3. Taxonomic Composition of Autochthonous Microbial Community of Dzhily-Su Hot Springs

Reads assigned to the bacterial domain accounted for 96.0% of the entire dataset. At the phylum level, the community was strongly dominated by Pseudomonadota (mean abundance across all samples was 82.2%), followed by Bacillota (6.9%), Cyanobacteriota (3.0%), Bacteroidota (1.9%), Verrucomicrobiota (1.8%), and Patescibacteria (1.4%) (Figure 2). The predominance of Pseudomonadota is in line with observations from other iron-carbonate mineral springs, where the water column is usually occupied by chemolithotrophic Fe(II)-oxidizing members of this phylum [7,16]. However, at a finer taxonomic resolution, a clear functional difference between the water-column and sediment fractions becomes obvious.

#### 2.3.1. Water-Column Fractions: Dominance of Iron-Oxidizing Gallionellaceae

Sequential filtration through 0.45 µm and 0.22 µm membranes was used to separate particle-associated and biofilm-forming microorganisms from the free-living and ultrasmall cell fractions, respectively, following the approach established for oligotrophic groundwater systems [17]. Consistent with this fractionation scheme, members of the family *Gallionellaceae* dominated the 0.45 µm retentate. These microorganisms form extracellular stalks and Fe(III) mineral aggregates that facilitate their retention on membranes with larger pore sizes [18]. *Gallionella* and *Sideroxydans*, the two principal neutrophilic Fe(II)-oxidizing genera of this family, were the most abundant across all four 0.45 µm samples (Figure 3). *Gallionella* reached 64.6% at the ‘eye’ outlet and 40.0% at the upper ‘joints’ outlet, while *Sideroxydans* peaked at 38.6% at the ‘stomach’ outlet. At the latter site, the combined abundance of *Gallionella*, *Sideroxydans*, and unclassified representatives of *Gallionellaceae* reached 80.2%, consistent with Fe(II)-oxidizing chemolithotrophs representing a major putative energy-yielding guild at this outlet. Members of the *Gallionellaceae* family are obligate or facultative microaerophilic chemolithotrophs that get energy from Fe(II) oxidation coupled to CO_2_ fixation via the Calvin cycle [1]. The physicochemical conditions at Dzhily-Su outlets (pH 6.22–6.74, Eh −34.9 to +74.8 mV, and O_2_ saturation 3.2–37.7%) correspond well to the established niche of neutrophilic FeOB [19].

The ‘liver’ outlet 0.45 µm fraction differed notably from the other three: *Gallionellaceae* were less abundant, while unclassified *Comamonadaceae* (36.7%) and *Ca.* Nitrotoga (6.3%) were prominent (Figure 3). *Ca.* Nitrotoga is a psychrotolerant nitrite-oxidizing betaproteobacterium whose activity depends on prior ammonia oxidation [20,21]. Its detection suggests that nitrification may represent an additional chemolithotrophic process at this outlet. The ‘liver’ spring also had the highest dissolved oxygen (37.7%) and TDS (4010 mg/L) among the sampled sites, conditions that may favor a broader range of chemolithotrophs relative to the more reducing outlets.

The 0.45 µm particle-associated fractions also harbored less abundant, but ecologically meaningful taxa, such as *Rhodoferax*, a psychrotolerant betaproteobacterium capable of either aerobic heterotrophy or dissimilatory Fe(III) reduction [22], and *Phreatobacter* (reaching 4.0% at the ‘eyes’ outlet), a genus, often associated with subsurface aquifers [23]. Their enrichment in the particle-associated fraction is compatible with the occurrence of microscale redox heterogeneity, which could facilitate the coexistence of facultative anaerobes and microaerophilic chemolithotrophs.

The 0.22 µm filter fraction from the ‘liver’ outlet showed a markedly different composition. Unclassified *Lysobacteraceae* dominated at 67.5%, whereas *Gallionella* and *Sideroxydans* together comprised only 2.5% (Figure 3). This shift is expected under the fractionation scheme applied: stalk-forming *Gallionellaceae* are efficiently retained by 0.45 µm membranes, and their near absence in the 0.22 µm fraction confirms that the two filtration steps capture distinct microbial guilds. The 0.22 µm fraction was also enriched in *Ca.* Omnitrophus (4.4%), an ultrasmall lineage belonging to the candidate phylum Omnitrophota. Their ultrasmall cell sizes allow free passage through 0.45 µm pores and subsequent enrichment on 0.22 µm filters [17].

#### 2.3.2. Sediment Fractions: Heterotrophic and Iron-Reducing Communities

Sediment microbial communities differed sharply from those in the spring water, showing a clear shift toward heterotrophic and facultatively anaerobic taxa (Figure 3). Sediment samples from the ‘liver’ and ‘joints’ outlets were strongly dominated by *Aeromonas* (65.0% and 57.4%, respectively) and *Shewanella* (9.4% and 17.8%, respectively), which collectively accounted for 74.4% and 75.2% of reads at these sites. Both genera are facultative anaerobes capable of dissimilatory Fe(III) reduction [24,25]. Their strong enrichment in the sediments is consistent with the potential for coupled iron transformations across the water–sediment interface. In such a scenario, Fe(III) minerals formed near the oxygenated water–sediment interface could serve as terminal electron acceptors for sediment-associated Fe(III)-reducing populations, with regenerated Fe(II) potentially becoming available for re-oxidation. Direct iron-speciation and process-rate measurements would be required to confirm this mechanism in situ [1,7].

*Proteiniclasticum* reached 11.2% in the sediment of the lower ‘joints’ spring. This genus comprises strictly anaerobic proteolytic fermenters of the order *Peptostreptococcales*, and its detection is consistent with fermentation of organic matter in deeper anoxic sediment horizons [26]. However, given the high recreational use of the ‘joints’ outlets and the incomplete removal of human-associated reads at this site (see Section 2.2.2), a contribution from allochthonous organic matter of anthropogenic origin cannot be excluded [27].

Cyanobacteria of the genera *Tychonema* CCAP 1459-11B and *Geminocystis* PCC-6308 were detected in the sediment fractions at low abundances (Figure 3). As both taxa typically form microbial mats, their occurrence suggests active phototrophic accretion at the sediment surface under shallow, light-penetrated water, rather than simple deposition from above. These organisms may also contribute to primary production and organic carbon input at the sediment-water interface.

The sediment sample from the upper ‘joints’ outlet showed completely different composition, with *Exiguobacterium* dominating at 33.1% while the abundance of *Aeromonas* was reduced to 8.2% (Figure 3). As noted in Section 2.2.2, this sample had the highest proportion of putatively allochthonous reads prior to filtering, and removal of over half the reads may have substantially altered the relative abundances of the remaining taxa. *Exiguobacterium* is a stress-tolerant gammaproteobacterium reported from geothermal springs, permafrost, and hypersaline lakes [28], making its occurrence in a warm mineral spring plausible. However, whether its dominance reflects the original community composition or an artifact of the in silico decontamination procedure remains uncertain.

#### 2.3.3. Archaeal Component and Candidate Phyla

The archaeal fraction (4.0% of total reads) was dominated by the phylum Nanoarchaeota (58.6% of archaeal reads). Nanoarchaeota are ultra-small ectosymbiotic archaea originally described from hyperthermophilic environments [29], although more recent studies have documented their occurrence in mesothermal and cold groundwater systems [30,31]. Their detection at the Dzhily-Su outlets (4–23 °C) is therefore not without precedent, though their ecological role in mesothermal iron-rich systems remains poorly understood.

Among the bacterial candidate phyla, members of the CPR superphylum were consistently detected across water samples at low but reproducible abundances (mean 1.4% across all samples). Additionally, *Candidatus* Omnitrophus (Omnitrophota phylum) reached 4.4% in the 0.22 µm fraction, enriched relative to the 0.45 µm fraction from the same outlet, consistent with its ultra-small cell size [32].

### 2.4. Alpha and Beta Diversity of the Autochthonous Microbial Community of the Dzhily-Su Springs

#### 2.4.1. Alpha Diversity

Alpha diversity metrics were calculated on the decontaminated dataset after rarefaction to the minimum sequencing depth across samples. Observed ASV richness ranged from 66 (sediment of the ‘eye’ outlet) to 213 (0.45 µm filter fraction of the ‘liver’ outlet). Across the eight samples, the Shannon index ranged from 1.73 to 3.12, and the Inverse Simpson index ranged from 2.29 to 7.58 (Table 3, Supplementary Table S2). The 0.45 µm fractions generally had the highest richness and diversity values, whereas the sediment samples had comparatively lower richness and diversity, consistent with the predominance of a small number of heterotrophic and iron-reducing genera, including *Aeromonas* and *Shewanella*.

Alpha diversity metrics did not differ significantly among sample fraction types (Kruskal–Wallis test, *p* > 0.05 for all metrics). Thus, despite pronounced differences in taxonomic composition between the water column and sediment communities, overall community complexity remained statistically comparable across the analyzed samples.

#### 2.4.2. Beta-Diversity and Correlation of Taxa with Environmental Parameters

Community composition, assessed using Bray–Curtis distance, differed significantly among sample fraction types (PERMANOVA; R^2^ = 0.53, F = 2.78, df = 2, *p* = 0.002). Within-group dispersion did not differ significantly among fraction types (PERMDISP; F = 1.81, df = 2, *p* = 0.279), indicating that the PERMANOVA result was not driven by unequal dispersion. This contrasts with the alpha-diversity patterns reported above, indicating that although overall community complexity was statistically comparable across fraction types, the identity and relative abundance of the constituent taxa differed markedly. A sensitivity analysis based on the original and taxon-filtered ASV tables yielded comparable water–sediment differentiation (PERMANOVA: R^2^ = 0.346, *p* = 0.016 and R^2^ = 0.358, *p* = 0.010, respectively; Supplementary Table S1), indicating that the principal community-level pattern was robust to the filtering procedure.

Results of the RDA constrained by temperature, pH, redox potential, and dissolved oxygen saturation are shown in Figure 4. The first two constrained axes accounted for 29.2% and 11.3% of the total variance in the Hellinger-transformed family-level community data, respectively. The water samples were positioned primarily along the positive part of RDA1 and were associated with *Gallionellaceae*, whereas the sediment samples were positioned predominantly along the negative part of RDA1 and were associated with *Aeromonadaceae* and *Shewanellaceae*. Redox potential and temperature were oriented toward the positive part of RDA1, while pH and dissolved oxygen saturation were oriented toward negative RDA2. However, the overall RDA model was not statistically significant (pseudo-F = 0.780, df = 4, *p* = 0.705), most likely because of the limited number of samples relative to the number of environmental predictors. The ordination is therefore presented as a descriptive visualization of community-environment associations rather than as evidence of statistically supported environmental control.

### 2.5. Taxonomy-Based Functional Predictions

Taxonomy-based functional prediction indicated contrasting distributions of iron-cycling categories among sample types. Reads assigned to aerobic chemolithotrophic iron oxidation were concentrated in the spring-water filter fractions, reaching the highest relative abundances at the stomach and eye outlets, consistent with the dominance of *Gallionellaceae* in these samples (Figure 5). In contrast, reads assigned to iron respiration were scarce in the filter fractions but accounted for approximately half of the community in two of the three sediment samples (‘liver’ and lower ‘joints’ outlets). This pattern reflected the abundance of *Aeromonas* and *Shewanella* in these sediments (Figure 3; Supplementary Table S3) and was consistent with the potential for coupled microbial iron cycling across the water–sediment interface.

Nitrogen-cycling functions showed a comparable but less pronounced contrast between water and sediments (Figure 5). Reads assigned to nitrification were most abundant at the liver outlet in both water fractions, consistent with the detection of the nitrite-oxidizing genus *Ca.* Nitrotoga. Reads assigned to nitrate reduction were concentrated primarily in the sediment samples, where it accounted for roughly a fifth to a quarter of the community.

Sulfur-related functions were assigned to a comparatively small fraction of the community across all samples. Reads assigned to dark sulfide/thiosulfate oxidation and sulfate reduction each remained below 0.5% at their respective maxima, indicating that sulfur-related functional assignments represented only a minor component of the predicted profile.

Sediment samples contained substantially higher proportions of reads assigned to chemoheterotrophy and fermentation, together accounting for approximately half of the predicted profile in each sediment sample. This pattern was consistent with the taxonomic dominance of facultatively anaerobic heterotrophs in these samples.

## 3. Discussion

### 3.1. A Canonical Neutrophilic Fe(II)-Oxidizing Consortium Under Magmatic-CO_2_ Overpressure

Water-column communities across the ferruginous Dzhily-Su outlets are strongly dominated by microaerophilic Fe(II)-oxidizing *Gallionellaceae*. *Gallionella* accounts for 64.6% of reads at the ‘eye’ outlet, whereas *Sideroxydans* reaches 38.6% at ‘stomach’. Together with unclassified *Gallionellaceae*, these two genera constitute 80.2% of the community at ‘stomach’ outlet. This structure closely mirrors the canonical microaerophilic Fe-oxidizing consortium documented at the Fuschna spring in the Swiss Alps, where *Gallionella* comprised ~99% of the FeOB fraction [7], and at the Bloomington iron-carbonate seep, where *Gallionella*, *Sideroxydans*, and *Aquabacterium* dominated planktonic samples [8,9]. The conservation of this planktonic *Gallionellaceae* signature across systems differing substantially in elevation and iron concentration suggests that circumneutral pH, microaerobic conditions, and the ferruginous character of the springs provide a broadly favorable ecological setting for neutrophilic FeOB, even under the unusual hydrogeochemical conditions. Dzhily-Su represents the highest-altitude neutrophilic Fe-carbonate spring complex where this consortium has been reported (2360 m a.s.l.) and the first documented case driven by a deep magmatic–metamorphic CO_2_ source [3]. Outlet-scale variation within the *Gallionellaceae* coincided with differences in temperature among the sampled outlets. *Gallionella* reaches its highest relative abundance at the cooler outlets (‘eye’ and ‘joints’ upper; 16.4–20.2 °C), whereas *Sideroxydans* peaks at the warmer outlets (‘stomach’ and ‘joints’; 22.4–22.6 °C). This alignment matches the reported growth temperature optima of isolate strains: 17–20 °C for *G.ferruginea* and ~30 °C for *S.lithotrophicus* [13,16,18]. Similar micro-scale partitioning was documented in South Wales coalfield aquifers, where relative proportions of these two genera reflected hydrogeological microenvironments rather than bulk fluid chemistry [13]. The correspondence between outlet temperature and the relative abundances of *Gallionella* and *Sideroxydans* suggests that temperature may contribute to genus-level niche partitioning across the sampled outlets. However, given the limited number of biological samples and the non-significant environmental RDA model, this relationship should be regarded as a hypothesis.

### 3.2. Water–Sediment Partitioning Consistent with Coupled Microbial Iron Cycling

The Dzhily-Su microbial landscape is characterized by striking compositional partitioning between the water column and underlying sediments. While water-column communities were enriched in microaerophilic *Gallionellaceae*, sediment samples were dominated by facultative gammaproteobacterial heterotrophs and genera containing characterized Fe(III)-reducing representatives: *Aeromonas* constitutes up to 65.0% of reads in ‘liver’ sediment and 57.4% in ‘joints’ sediment, whereas *Shewanella* accounts for 9.4–17.8% across sediment sites. This spatial partitioning closely resembles the pattern documented at the Bloomington groundwater seep, where planktonic *Gallionella* and *Sideroxydans* were spatially juxtaposed with sedimentary *Aeromonas*, *Rhodoferax*, and *Geobacter* [8,9]. At Bloomington, *Aeromonas* acted as a facultative Fe(III) reducer, coupling the oxidation of organic carbon to the reduction in poorly crystalline Fe(III) (oxyhydr)oxides on centimeter scales and sustaining an active microbial “ferrous wheel” [9]. In comparison, the water–sediment divergence at Dzhily-Su is remarkably pronounced (PERMANOVA R^2^ = 0.53, F = 2.78, *p* = 0.002; PERMDISP *p* = 0.279), matching or exceeding the differentiation reported for Bloomington and subarctic tundra pools [12]. This taxonomic partitioning is consistent with a biogeochemical organization analogous to that described at the Bloomington groundwater seep. However, unlike in the Bloomington system, direct Fe(II)/Fe(III) transformation rates were not measured at Dzhily-Su; therefore, the presence of an active microbial ‘ferrous wheel’ remains a biologically plausible interpretation rather than a directly demonstrated process.

Unlike in Alaskan tundra pools [12] or the Bloomington seep, where *Rhodoferax* serves as a key iron-reducing taxon, its relative abundance at Dzhily-Su is low. Instead, *Aeromonas* was the dominant sediment-associated genus containing well-characterized Fe(III)-reducing representatives, suggesting that taxonomically distinct assemblages may occupy functionally analogous niches. The iron-oxidizing counterpart of this system also differs from typical models. Dzhily-Su sediment communities lack notable *Leptothrix* or *Zetaproteobacteria* populations, despite ubiquitous ochreous deposits. The low relative abundance of *Leptothrix* may be associated with the rapidly flowing, spatially restricted outlet habitats, which differ from the more diffuse iron-rich environments where sheath-forming mixotrophic FeOB are commonly reported [10]. In turn, the lack of typical marine microaerophiles *Zetaproteobacteria* is rather expected in continental springs [33]. Thus, the Dzhily-Su assemblage is compatible with a putative coupled microbial iron cycle functionally analogous to the ‘ferrous wheel’ described at Bloomington, although direct functional validation will be required.

### 3.3. Limited Representation of Alternative Fe-Cycling Lineages

Taxa associated with alternative Fe-cycling pathways that are prominent in other neutrophilic or weakly acidic systems are either absent or reduced to background levels at Dzhily-Su. Lineages inferred to drive photoferrotrophy or nitrate-dependent Fe(II) oxidation, which co-occur alongside *Zetaproteobacteria* in marine and estuarine sediments [33], were not recovered above baseline relative abundance. Canonical photoferrotrophic genera (*Chlorobium*, *Rhodobacter*) and nitrate-reducing FeOB (*Hoeflea*, *Dechloromonas*) are absent. This taxonomic signature is consistent with the dynamic, shallow, and highly flushed hydrology of the outlet channels, which may limit the development of stable light-anoxia stratification required by anoxygenic photoferrotrophs. Furthermore, ambient nitrate concentrations in the recharge waters of the Elbrus volcanic cluster remain extremely low [3]. The prevailing pH range (6.22–6.74) also lies above the acidic window (optimal at pH ~4.6) where rapid abiotic Fe(II)-driven nitrate reduction to N_2_O becomes significant [34]. The detection of *Ca.* Nitrotoga at the liver outlet indicates the presence of a bacterial lineage containing characterized nitrite oxidizers, although the activity and quantitative importance of nitrification were not determined.

Heavy-metal-tolerant, acidophilic *Gallionellaceae* lineages, such as those described in acid mine drainage [35] are likewise absent. Fluids at Dzhily-Su are circumneutral and lack toxic metal loading despite elevated electrical conductivity. Thus, the *Gallionellaceae* assemblage aligns compositionally with pristine analogs such as Fuschna and Bloomington rather than metal-impacted systems.

Finally, in contrast to the ferromanganese sediments of Lake Baikal, the primary regional reference for Russian Fe-cycling systems [36], Dzhily-Su deposits lack dominant Chloroflexota, Acidobacteriota, and Methylomirabilota communities linked to anaerobic methane oxidation (AOM). This contrast may reflect fundamental hydrogeological differences: Lake Baikal Fe-Mn horizons form within deep, buried sediments under ~1000 m of water, whereas iron-rich precipitates form rapidly upon atmospheric exposure at Dzhily-Su spring outlets. Under these conditions, AOM-associated archaea (*Methanoperedenaceae*) and organoheterotrophic anaerobes fail to establish stable populations. This contrast suggests that fluid residence time and the persistence of redox-stratified microhabitats may contribute to the architecture of neutrophilic Fe-cycling communities.

### 3.4. Candidate Divisions and Archaea: An Unexpected Complexity

The analysis of the fine-size fraction (0.22 µm filtrate following 0.45 µm filter) revealed a notable representation of poorly characterized candidate lineages and archaea. In this fraction, Nanoarchaeota constitute up to 58.6% of the archaeal reads (~4.0% of total reads in the 0.22 µm fraction), *Ca.* Omnitrophus reaches 4.4%, and members of the Patescibacteria are consistently detected at percent-level relative abundances. This enrichment aligns with the ultra-small cell sizes characteristic of DPANN archaea and CPR bacteria. A comparable association was reported in South Wales coalfield aquifers, where Omnitrophicaeota and Patescibacteria collectively constituted up to 37% of the community at Betaproteobacteriales-rich sites and were inferred to act as epibionts or symbionts of dominant Fe-oxidizing bacteria [13]. The presence of these lineages at Dzhily-Su raises the possibility that similar host–parasite or host–symbiont interactions may occur within deeply sourced, magmatically driven carbonate springs. Although Nanoarchaeota are historically associated with high-temperature environments, representatives of this phylum have been recently documented in mesophilic groundwaters [31,37]. In such ambient-temperature Fe-rich systems, their host spectrum remains largely uncharacterized and likely involves distinct mesophilic partners. However, further corroboration of this hypothesis will require shotgun metagenomic analysis.

### 3.5. Study Limitations and Future Directions

The present study has several limitations that should be considered when interpreting the observed ecological patterns. The relatively small number of biological samples and the uneven representation of sample fractions limit the statistical power of comparisons across habitats and environmental variables. Although the sampling design included water and sediment samples collected across multiple outlets, no replicate samples were taken from the same compartment within individual outlets. Consequently, the observed patterns are site-specific and hypothesis-generating. In addition, all samples analyzed here were collected during a single field campaign in August 2022; therefore, the seasonal variation remains unstudied. Seasonal sampling will be required to determine whether the water–sediment partitioning observed during this campaign persists under different hydrological and physicochemical conditions.

## 4. Methods and Materials

### 4.1. Sampling Procedures

The Dzhily-Su mineral spring complex is located on the northern slope of Mt. Elbrus, in the upper reaches of the Malka River, Kabardino-Balkarian Republic, Russia, at 2350–2380 m above sea level [3,4,6]. In August 2022, water and sediment samples were collected from all five accessible outlets with active discharge identified within the local spring complex. These outlets are informally named ‘liver’, ‘stomach’, ‘eye’, and ‘joints’ (lower and upper outlets) according to their traditional balneological use by local residents. The sampling sites were therefore not intended to constitute an evenly spaced spatial transect but to represent the distinct active discharge outlets present within the study area. Temperature, pH, redox potential (Eh), conductance, and dissolved oxygen saturation were measured in situ using a YSI ProDSS Multiparameter Digital Water Quality Meter (YSI Incorporated, Yellow Springs, OH, USA). Coordinates were recorded with a handheld GPS receiver.

For microbial community analysis, 5–10 L of water per outlet were sequentially filtered through 0.45 µm and 0.22 µm Sterivex™ filter units (MerckKGaA, Darmstadt, Germany) using a peristaltic pump, to separate particle-associated/biofilm-forming and free-living/ultrasmall-cell fractions [38]. Sediment samples were collected into sterile 5 mL Eppendorf Safe-Lock microcentrifuge tubes (Eppendorf SE, Hamburg, Germany) using sterile spatulas. All samples were fixed with RNAlater immediately after collection and stored at 4 °C until they were transferred to the laboratory (approximately one week).

In total, two environmental samples (water and sediment) were collected for each available outlet. Accordingly, the sampling design was intended to provide an initial site-specific characterization of microbial communities across the active outlets, rather than to quantify distance-dependent spatial patterns or within-outlet biological variability. Technical library PCR replicates were used to assess and improve analytical reproducibility but were not treated as independent biological replicates.

### 4.2. DNA Extraction, 16S rRNA Amplicon Library Preparation and Sequencing

Total genomic DNA was extracted from Sterivex™ filter membranes and from sediment samples using the DNeasy PowerLyzer PowerSoil Kit (Qiagen GmbH, Hilden, Germany) according to the manufacturer’s instructions. Filter membranes were aseptically excised prior to lysis. Mechanical cell disruption was performed using a FastPrep-24 5G homogenizer (MP Biomedicals, Solon, OH, USA) in PowerBead tubes (Qiagen, Germany) for 5 min. Final DNA purification was carried out on a QIAcube Connect automated platform (Qiagen, Germany). Sterile water processed in parallel with the samples through all extraction steps served as a negative extraction control. DNA purity was assessed spectrophotometrically (A260/A280 and A260/A230 ratios) using a NanoDrop 2000 spectrophotometer (Thermo Fisher Scientific Inc., Waltham, MA, USA), and DNA concentration was determined fluorometrically using a Qubit 4.0 Fluorometer (Thermo Fisher Scientific, USA).

The V4 region of the prokaryotic 16S rRNA gene was amplified using a two-step PCR protocol [39]. The first-step primers were target-specific, partially corresponded to the universal primer pair 515F/Pro-mod-805R [40] and contained Illumina-compatible overhangs [39]. Four variants of each primer carrying heterogeneity spacers of different lengths were combined in equimolar proportions to increase nucleotide diversity during the initial sequencing cycles [41].

The first PCR was performed in duplicate for each DNA sample in a total volume of 25 µL containing 5 µL of 5× qPCRmix-HS SYBR master mix (Evrogen, Moscow, Russia), 0.25 µL of each 25 µM primer pool, 1 µL of template DNA, and nuclease-free water. Amplification was performed on a CFX96 Real-Time PCR Detection System (Bio-Rad, Hercules, CA, USA) using the following cycling program: initial denaturation at 95 °C for 2 min; 25 cycles of 95 °C for 25 s, 56 °C for 20 s, and 72 °C for 30 s; and final extension at 72 °C for 5 min.

Depending on the amplification curves, first-round products were diluted two- or five-fold and 4 µL of the diluted product was used as template in the second PCR. This indexing PCR was performed in a total volume of 30 µL containing 6 µL of 5× qPCRmix-HS SYBR master mix, 3 µL each of 5 µM i5 and i7 indexing primers, 4 µL of diluted first-round product, and nuclease-free water. The cycling program consisted of initial denaturation at 95 °C for 2 min; 8 cycles of 95 °C for 20 s, 59 °C for 20 s, and 72 °C for 30 s; and final extension at 72 °C for 5 min.

The amplicon libraries were purified using a QIAquick Gel Extraction Kit (Qiagen, Germany), and checked using a Bioanalyzer 2100 (Agilent Technologies, Santa Clara, CA, USA). The final libraries were pooled in equimolar amounts and sequenced on an Illumina MiSeq platform in paired-end mode (2 × 156 bp).

### 4.3. Processing and Taxonomic Classification of Sequencing Reads

Adapter sequences were removed using cutadapt v3.5 [42]. Quality filtering, denoising, paired-end read merging, chimera removal, and inference of Amplicon Sequence Variants (ASVs) were performed using the DADA2 pipeline v1.34.0 in R v4.4.0 [43]. One water sample (0.22 µm fraction, ‘stomach’ outlet) was excluded prior to ASV inference due to insufficient sequencing depth (4653 reads), which precluded reliable denoising. Taxonomic classification of ASVs was performed against the SILVA reference database, release 138.2 [44].

### 4.4. Decontamination of Human-Associated Allochthonous Taxa

Preliminary taxonomic screening revealed elevated proportions of reads assigned to genera characteristic of the human gut microbiome (e.g., *Klebsiella*, *Escherichia-Shigella*, *Ewingella*) in one sediment sample from the most heavily bathed outlet. This finding is consistent with the well-documented tendency of fecal indicator bacteria to associate with particulate matter and accumulate in sediments of recreational water bodies [45]. It is noteworthy that none of these taxa have been identified as dissimilatory iron-reducing or iron-oxidizing bacteria, which are capable of sustaining autochthonous populations under the physicochemical conditions of cold, Fe-rich mineral springs [46]. In order to reduce noise resulting from recreational and anthropogenic contamination and to enhance the autochthonous signal, reads assigned to the genera *Klebsiella*, *Escherichia*-*Shigella*, *Ewingella*, *Bacteroides*, *Bifidobacterium*, *Faecalibacterium*, *Blautia*, *Alistipes*, *Lactobacillus*, *Akkermansia*, and *Ruminococcus* were removed from the ASV table prior to all downstream community and statistical analyses. The proportion of reads removed per sample is reported in the Results section (Table 2). The subsequent analysis was conducted on the decontaminated dataset. To evaluate the influence of taxon-based filtering, Bray–Curtis dissimilarities, PERMANOVA, and PERMDISP were additionally calculated for the original, unfiltered ASV table. The resulting community pattern and effect estimates were compared with those obtained from the taxon-filtered dataset.

### 4.5. Alpha- and Beta-Diversity Analysis

Prior to alpha diversity estimation, the decontaminated ASV table was rarefied to the minimum sequencing depth observed across the eight samples using the rarefy_even_depth function of the phyloseq package v1.50.0 [47]. Alpha diversity metrics were calculated using the estimate_richness function of phyloseq v1.50.0. The differences in each index between the sample fraction types (0.22 µm filter, 0.45 µm filter, sediment) were assessed using the non-parametric Kruskal–Wallis test.

Beta diversity was assessed on the rarefied, decontaminated ASV table using Bray–Curtis dissimilarity, calculated with the vegdist function of the vegan package v2.7.3 [48]. Homogeneity of within-group multivariate dispersions was tested using betadisper followed by a permutation test (permutest, 999 permutations), and differences in community composition between sample fraction types were tested by permutational multivariate analysis of variance (PERMANOVA) using the adonis2 function (999 permutations). To relate community structure to physicochemical parameters, ASV abundances were Hellinger-transformed (decostand, method = “hellinger”) [49], and redundancy analysis (RDA) was performed at the family level using the rda function of vegan, constrained by temperature, pH, redox potential, and dissolved oxygen saturation. The statistical significance of the overall RDA model and of individual ordination axes was assessed using anova.cca (999 permutations).

### 4.6. Taxonomy-Based Prediction of Microbial Functional Potential

The predicted functional profile of the microbial community was inferred by mapping taxonomic assignments onto metabolic functional groups using the FAPROTAX database v.1.2.12 [50]. The standard FAPROTAX ‘iron_respiration’ category contains predominantly species-level associations. Consequently, ASVs classified only as *Aeromonas* or *Shewanella* were not assigned to this category, although both genera include experimentally characterized Fe(III)-reducing representatives [24,51,52]. To capture this potential at the taxonomic resolution available in the present dataset, *Aeromonas* and *Shewanella* were included through a custom genus-level assignment to the iron_respiration category. Functional-category abundances were expressed as percentages of total reads per sample. These assignments represent hypothesis-generating estimates of taxonomy-based functional potential and do not demonstrate the presence or expression of specific functional genes or in situ metabolic activity. In particular, the custom genus-level assignment does not imply that every detected *Aeromonas* or *Shewanella* ASV is capable of Fe(III) reduction.

## 5. Conclusions

This study provides the first cultivation-independent molecular characterization of the high-altitude Dzhily-Su ferruginous mineral spring complex on Mt. Elbrus. Despite the unusual geological setting, including magmatic–metamorphic CO_2_ input, high elevation, and elevated conductivity, the spring water communities contained a canonical assemblage of neutrophilic Fe(II)-oxidizing taxa dominated by *Gallionella* and *Sideroxydans*. Their distribution among outlets suggests possible niche partitioning associated with local physicochemical conditions, although the limited sample size precludes assigning individual environmental variables a causal role. Water-column communities enriched in putative Fe(II) oxidizers were compositionally distinct from sediment communities enriched in *Aeromonas* and *Shewanella*, taxa that include well-characterized Fe(III)-reducing representatives. This complementary distribution is consistent with the potential for a coupled microbial iron cycle across the water–sediment interface but does not directly demonstrate in situ iron-transforming activity. Fine-size fractions additionally contained poorly characterized lineages, including Patescibacteria, *Ca.* Omnitrophus, and Nanoarchaeota. Future replicated and seasonal sampling, direct Fe(II)/Fe(III) speciation, and metagenomic or activity-based analyses will be required to test the ecological mechanisms suggested by these taxonomic patterns and to resolve the metabolic capabilities of the detected lineages.

## Figures and Tables

**Figure 1:**
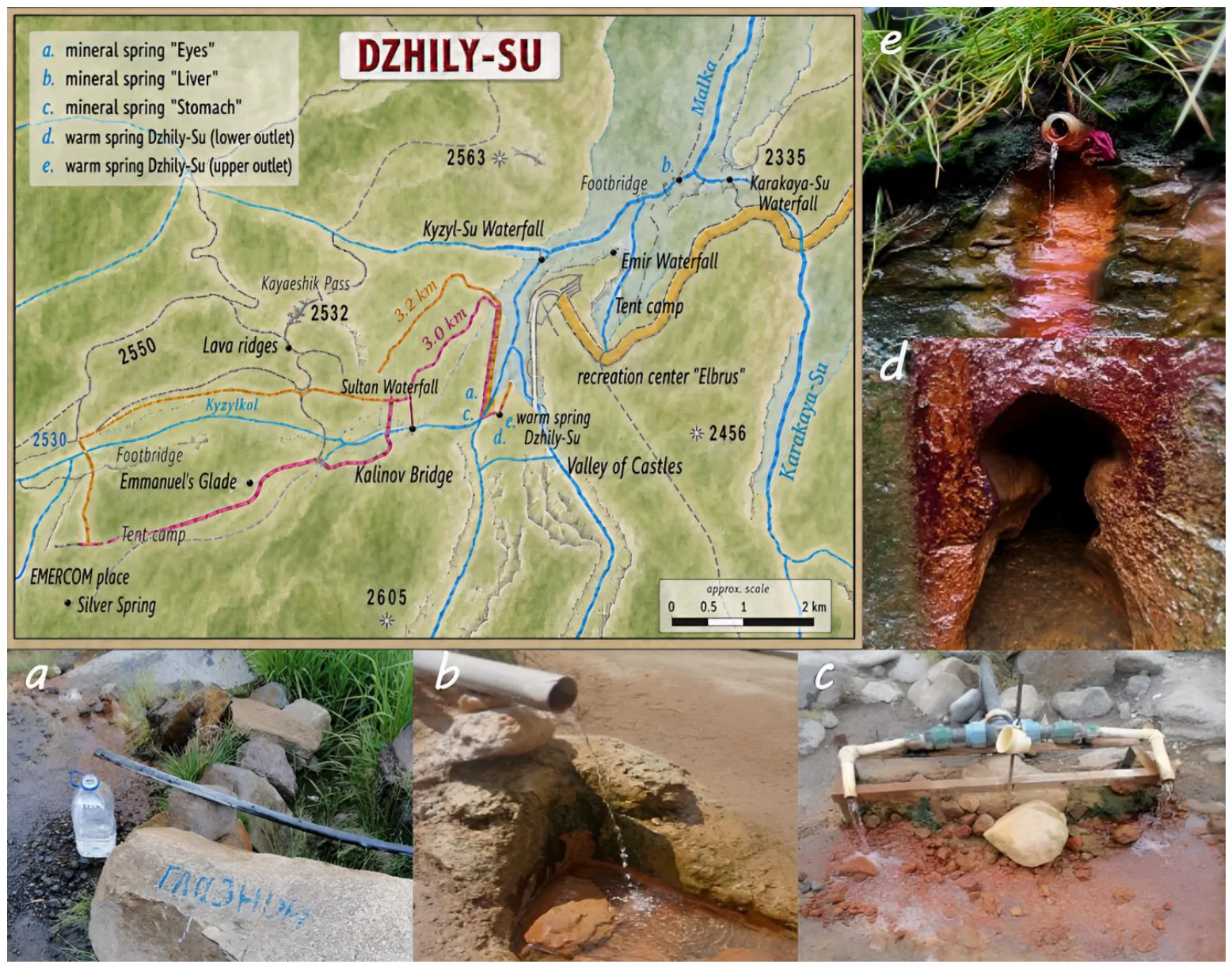
Location and field appearance of the Dzhily-Su mineral spring complex. The inset map shows the sampling area on the northern slope of Mt. Elbrus (Kabardino-Balkaria, Russia), with the five sampled outlets indicated: (**a**) mineral spring ‘eyes’; (**b**) mineral spring ‘liver’; (**c**) mineral spring ‘stomach’; (**d**) warm spring Dzhily-Su, lower outlet (‘joints’); (**e**) warm spring Dzhily-Su, upper outlet (‘joints’). Field photographs illustrate the characteristic morphology of each outlet: (**a**) the ‘eyes’ spring, fed by a submerged pipe discharging into a stone basin; (**b**) the ‘liver’ spring, discharging through a natural rock channel with ochre-colored iron hydroxide precipitate; (**c**) the ‘stomach’ spring, equipped with a tap and trough, with prominent orange ferruginous sediment; (**d**) the lower Joints outlet, exhibiting extensive dark rust-colored iron oxide encrustations around the discharge pipe; (**e**) the upper Joints outlet, showing the most intense orange-red ferruginous biofilm and mineral precipitate.

**Figure 2:**
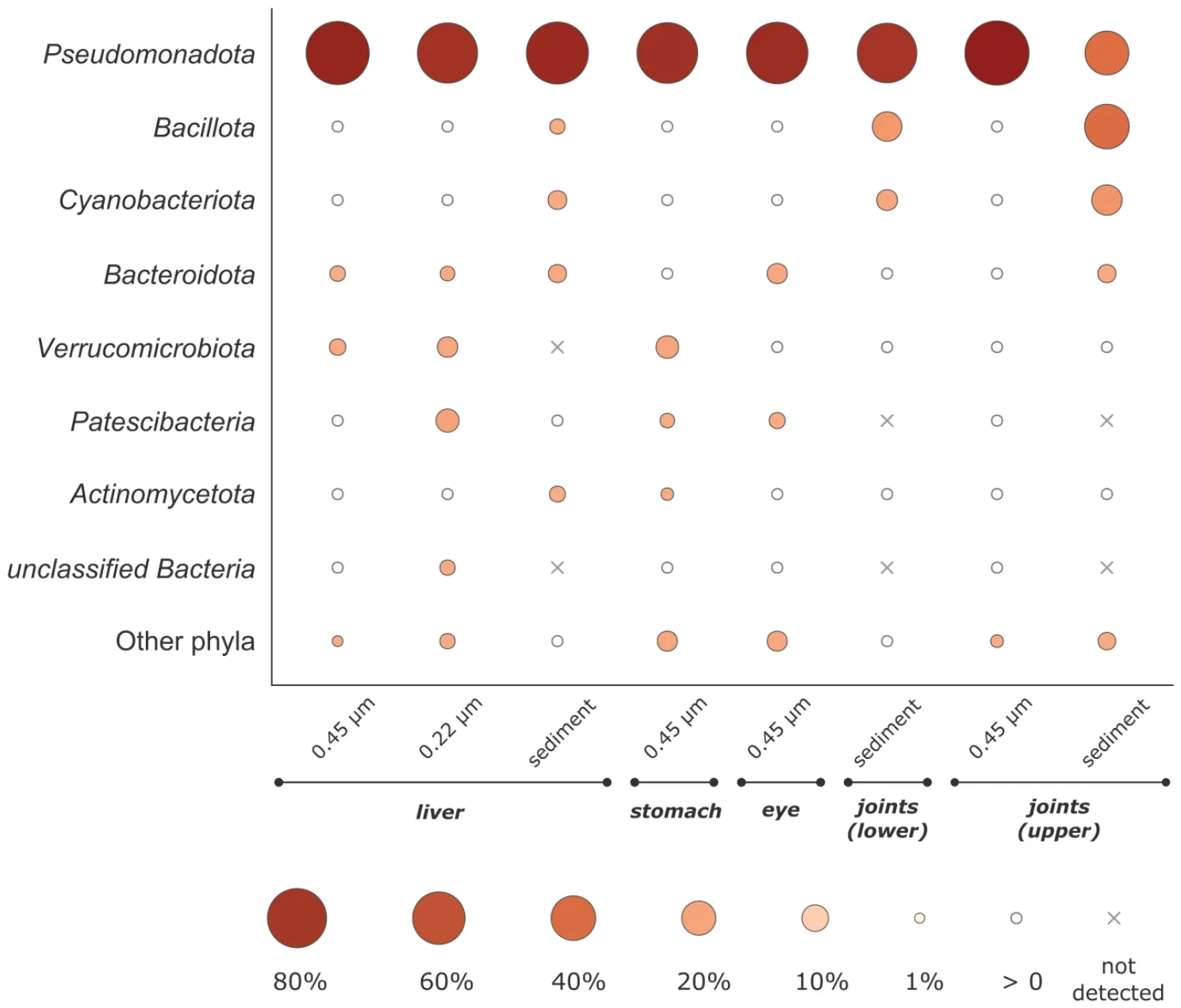
Phylum-level taxonomic composition of the Dzhily-Su microbial communities after removal of recreationally associated taxa. Both circle size and fill-color intensity represent relative abundance (%), with larger and darker circles indicating higher relative abundance. Open circles indicate taxa detected at <1% relative abundance, whereas crosses indicate taxa not detected. The eight most abundant phyla are shown, and the remaining phyla are grouped as “Other phyla”. Sample fractions are indicated below each column.

**Figure 3:**
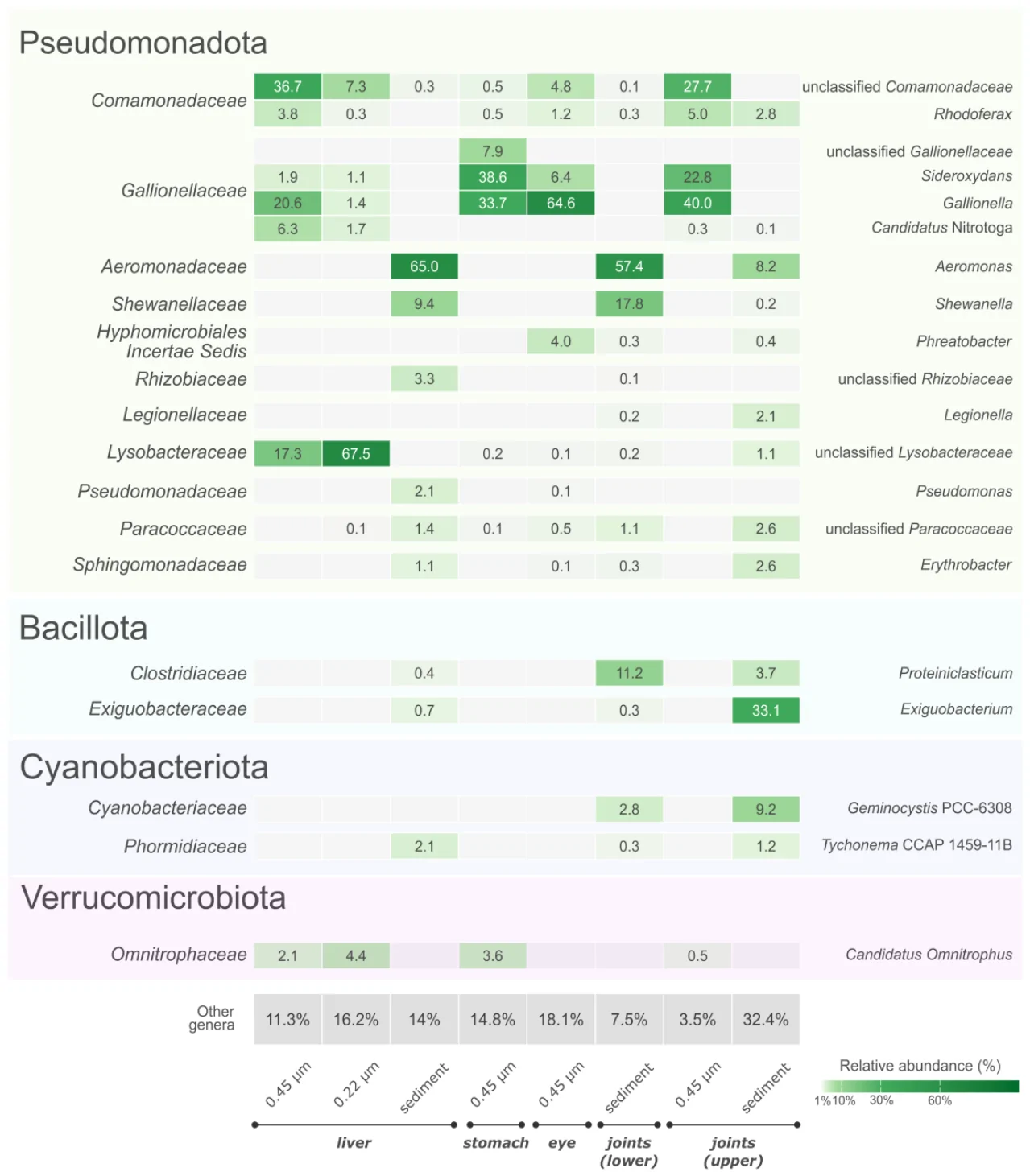
Genus-level taxonomic composition of the autochthonous microbial community of Dzhily-Su mineral springs. Heatmap shows the relative abundance (%) of the 20 most abundant genera after removal of recreational contaminants, grouped by family and phylum. Genera with relative abundance below 0.1% in a given sample are not labelled. The percentage of reads assigned to genera not included in the heatmap is shown in the “Other genera” row.

**Figure 4:**
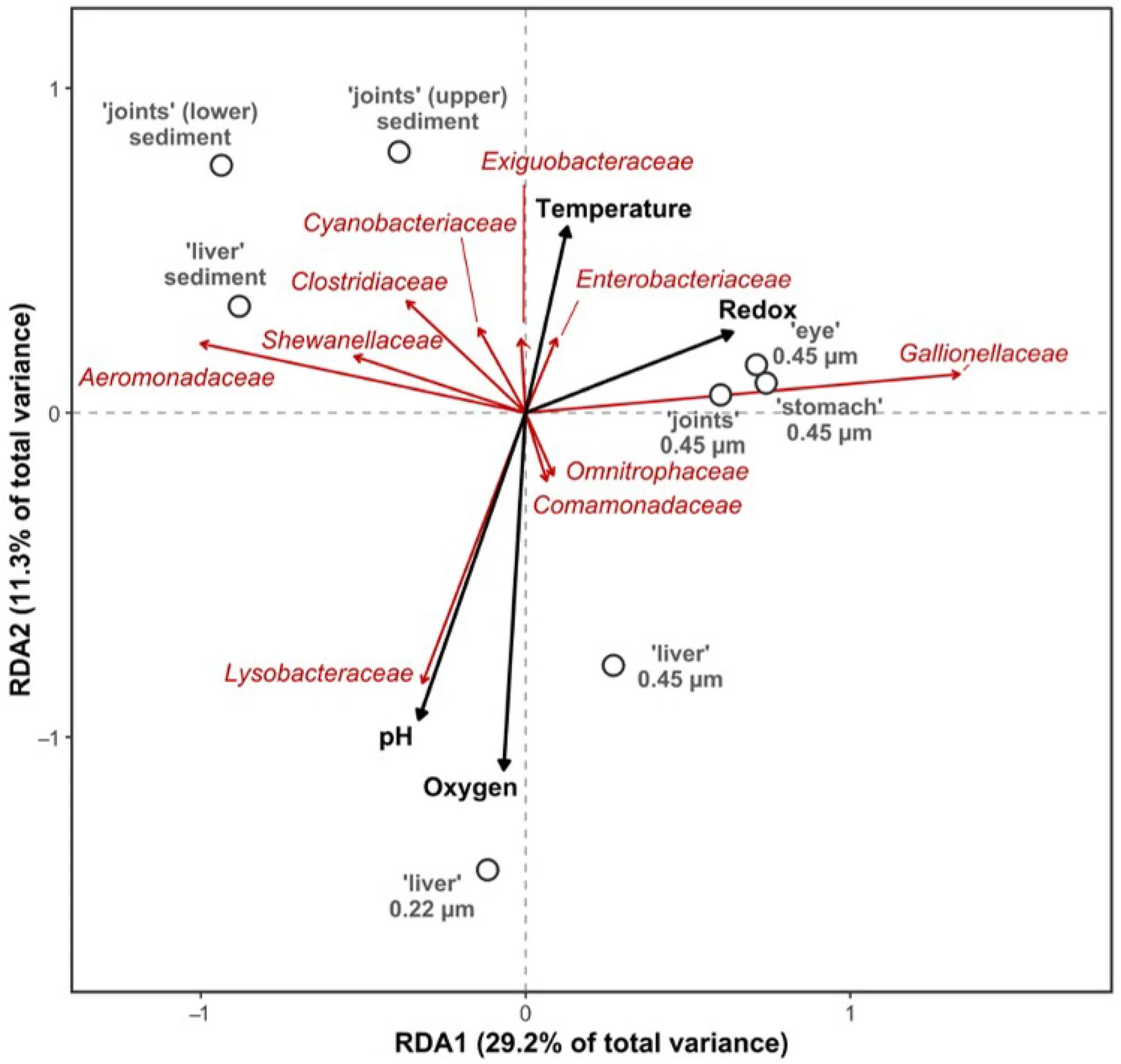
Redundancy analysis (RDA) of Hellinger-transformed family-level microbial community composition constrained by temperature, pH, redox potential, and dissolved oxygen saturation. Open circles represent individual microbial-community samples. Red arrows indicate the ten bacterial families contributing most strongly to the first two constrained axes, and black arrows indicate the physicochemical variables. Sample labels indicate the spring outlet and sample type. The low-depth stomach 0.22 µm filter sample was excluded before analysis.

**Figure 5:**
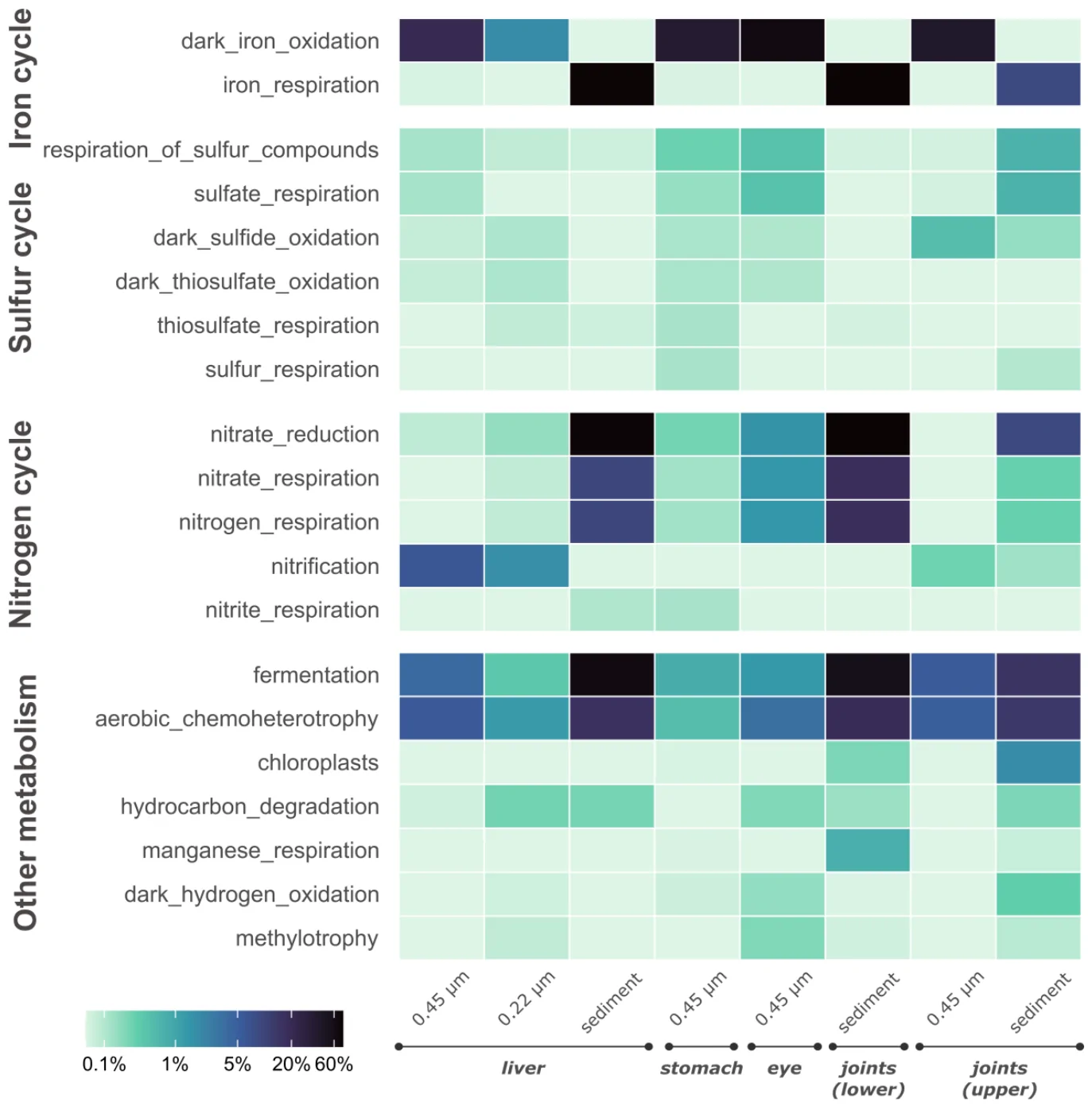
Taxonomy-based prediction of microbial functional potential using FAPROTAX. Values used for heatmap represent the percentage of total reads taxonomically assigned to each category and do not represent directly measured metabolic activity. Cell color indicates the untransformed relative abundance, displayed using a pseudo-logarithmic color scale to improve visualization across the broad abundance range. Color-scale labels show the corresponding relative-abundance percentages.

**Table 1:** Physicochemical parameters of the Dzhily-Su sampling sites.

Local Name	Sample IDs	Coordinates	T (°C)	pH	TDS (mg/L)	Eh (mV)	O_2_ (% Sat.)
Liver	0515	43.4334° N, 42.5348° E	16.5	6.74	4010	−23.2	37.7
Stomach	0516A	43.4338° N, 42.5360° E	22.4	6.54	2543	−31.3	17.9
Eye	0516B	43.4340° N, 42.5360° E	20.2	6.22	720	74.8	16
Joints (lower outlet)	0516D	43.43379° N, 42.53585° E	22.6	6.36	2564	−34.9	3.2
Joints (upper outlet)	0516E	43.43373° N, 42.53604° E	16.4	6.41	1997	8	11.9

**Table 2:** Relative abundance (%) of recreationally associated genera excluded from the dataset ^1^.

Taxon	0515B_sed	0516D_sed	0516E_sed	0.22 µm Filters (Mean)	0.45 µm Filters (Mean)
*Klebsiella*	1.28	0.05	42.26	0	0
*Escherichia–Shigella*	0	0.83	13.89	0.05	0.03
*Ewingella*	0	0.15	6.87	0	0
*Bacteroides*	0.43	0.91	2.5	0.09	0.09

^1^ Values represent percentages of total reads before decontamination. Only genera reaching ≥1% relative abundance in at least one sample or sample group are shown.

**Table 3:** Alpha diversity indices of the autochthonous microbial community across sample types ^1^.

Sample Type	*n*	Observed	Chao1	Shannon	Inverse Simpson
Filter 0.22 µm ^2^	1	178	189.9	2.25	3.34
Filter 0.45 µm	4	148 ± 50	160.2 ± 46	2.40 ± 0.25	5.16 ± 0.70
Sediment	3	93 ± 31.1	93.3 ± 30.8	2.19 ± 0.80	4.21 ± 2.93

^1^ Values are mean ± SD after rarefaction to the minimum sequencing depth. Single Filter 0.22 µm sample has no SD (*n* = 1). Kruskal–Wallis test across fraction types: Observed *p* = 0.213, Chao1 *p* = 0.132, Shannon *p* = 0.683, Inverse Simpson *p* = 0.499. ^2^ The 0.22 µm category is represented by a single sample (*n* = 1); therefore, statistical comparisons involving this category have limited inferential value and should be interpreted cautiously.

## Data Availability

All the related data, including sample description and sequencing reads were deposited into the China National GeneBank DataBase (https://db.cngb.org/(accessed on 20 September 2026)) under the Project ID CNP0009703.

## Supplementary Materials

The following supporting information can be downloaded at https://www.mdpi.com/article/10.3390/ijms27198549/s1.

## Abbreviations

The following abbreviations are used in this manuscript:

AOM	Anaerobic methane oxidation
a.s.l.	Above sea level
ASV	Amplicon sequence variant
DOC	Dissolved organic carbon
Eh	Redox potential
FeOB	Fe(II)-oxidizing bacteria
FeRB	Fe(III)-reducing bacteria
PERMANOVA	Permutational multivariate analysis of variance
RDA	Redundancy analysis
SpC	Specific conductance
TDS	Total dissolved solids
