# Supplementary material for: Water–Sediment Partitioning of a Neutrophilic Iron-Cycling Microbiome in the High-Altitude Dzhily-Su Mineral Springs (Mt. Elbrus)
Source: Int J Mol Sci. 2026 Sep 24;27(19):8549. doi: 10.3390/ijms27198549 (PMC13664883; doi:10.3390/ijms27198549)
Supplement: Supplementary file 1 [file ijms-27-08549-s001.zip › ijms-4558452-supplementary.pdf]

SUPPLEMENTARY MATERIALS

SUPPLEMENTARY TABLES

Supplementary Table S1. Read processing statistics.

	input	filtered	denoisedF	denoisedR	merged	non-chimeric
0515A_0-22.repl1	27568	24637	24413	24515	24348	23976
0515A_0-22.repl2	27884	25990	25775	25801	25660	25000
0515A_0-45.repl1	45843	41314	41089	41161	40991	38914
0515A_0-45.repl2	41134	32527	32362	32408	32293	30607
0515B_sed.repl1	30336	27171	27147	27144	27125	25994
0515B_sed.repl2	36896	32342	32279	32282	32231	30382
0516A_0-22.repl1	3684	3150	2965	3089	2930	2607
0516A_0-22.repl2	969	806	796	788	779	698
0516A_0-45.repl1	37180	34160	33725	33932	33595	31254
0516A_0-45.repl2	45909	41364	41090	41160	40964	37828
0516B_0-45.repl1	46762	42531	42168	42346	42066	38937
0516B_0-45.repl2	18867	17376	17328	17294	17250	10962
0516D_sed.repl1	37010	30291	30240	30234	30189	28058
0516D_sed.repl2	43008	38953	38863	38853	38782	36174
0516E_0-45.repl1	52710	45665	45475	45580	45430	42151
0516E_0-45.repl2	46972	42221	42036	42125	41986	39684
0516E_sed.repl1	30404	28249	28153	28177	28106	27043
0516E_sed.repl2	52267	47553	47246	47367	47130	45052

Supplementary Table S2. Individual alpha-diversity metrics per sample.

sample	sample_type	Observed	Chao1	Shannon	InvSimpson
0515A_0-22	Filter_0.22	178	189.875	2.247631	3.337966778
0515A_0-45	Filter_0.45	124	141	2.43083	5.565487132
0515B_sed	Sediment	66	66.75	1.733113	2.2924496
0516A_0-45	Filter_0.45	213	220.6667	2.635475	5.890619942
0516B_0-45	Filter_0.45	158	166.6667	2.484853	4.34558057
0516D_sed	Sediment	86	86.1	1.731259	2.754259853
0516E_0-45	Filter_0.45	97	112.5455	2.040117	4.833941065
0516E_sed	Sediment	127	127	3.118842	7.581155547

Supplementary Table S3. Full FAPROTAX functional annotation matrix*.

Process	0515A_0-22	0515A_0-45	0515B_sed	0516A_0-45	0516B_0-45	0516D_sed	0516E_0-45	0516E_sed
methanotrophy	0	0	0	0	0.24	0	0	0
methanol_oxidation	0.07	0	0	0	0	0.03	0	0.09
methylotrophy	0.07	0	0	0	0.24	0.03	0	0.09
aerobic_nitrite_oxidation	1.68	6.26	0	0	0	0	0.3	0.15
nitrification	1.68	6.26	0	0	0	0	0.3	0.15
sulfate_respiration	0	0.13	0	0.18	0.5	0	0.02	0.74
sulfur_respiration	0	0	0	0.13	0	0	0	0.1
thiosulfate_respiration	0.07	0	0.04	0.13	0	0.02	0	0
respiration_of_sulfur_compounds	0.07	0.13	0.04	0.31	0.5	0.02	0.02	0.74
arsenate_respiration	0	0	0	0.13	0	0	0	0
dissimilatory_arsenate_reduction	0	0	0	0.13	0	0	0	0
knallgas_bacteria	0.04	0	0	0	0	0.01	0	0.19
dark_hydrogen_oxidation	0.04	0	0	0.04	0.19	0.01	0	0.35
nitrate_ammonification	0	0	0	0.13	0	0	0	0
nitrite_ammonification	0	0	0	0.13	0	0	0	0
nitrite_respiration	0	0	0.11	0.13	0	0	0	0
cellulolysis	0	0	0	0	0	0	0	0.09
dark_sulfide_oxidation	0.12	0.06	0	0.12	0.11	0	0.55	0.18
dark_sulfur_oxidation	0	0	0	0	0	0	0	0
dark_thiosulfate_oxidation	0.12	0.06	0	0.12	0.11	0	0	0
dark_oxidation_of_sulfur_compounds	0.12	0.06	0	0.12	0.11	0	0.55	0.18
manganese_respiration	0	0	0	0.01	0	0.79	0	0.05
fermentation	0.41	3.95	65.66	0.84	1.39	58.55	5.57	15.27
aerobic_chemoheterotrophy	1.3	5.72	16.48	0.54	3.42	19.22	5.13	13.26
human_gut	0.01	0.01	0	0.08	0	0.01	0.02	0
human_associated	0.01	0.01	0.5	0.08	0.01	0.01	0.02	0
mammal_gut	0.01	0.01	0	0.08	0	0.01	0.02	0
animal_parasites_or_symbionts	0.01	0.01	0.5	0.08	0.01	0.01	0.02	0
aromatic_compound_degradation	0	0	0	0	0.05	0	0.05	0.09
dark_iron_oxidation	1.79	20.55	0	36.69	65.43	0	40.81	0
iron_respiration	0	0.02	74.42	0.01	0	75.23	0	8.61
nitrate_respiration	0.07	0	9.56	0.14	1.42	17.84	0	0.33
nitrate_reduction	0.19	0.08	74.57	0.29	1.54	75.26	0	8.75
nitrogen_respiration	0.07	0	9.56	0.14	1.42	17.84	0	0.33
fumarate_respiration	0	0	0	0.13	0	0	0	0
intracellular_parasites	0.12	0.02	0	0.22	0.01	0.24	0.01	2.45
predatory_or_exoparasitic	0.04	0	0	0	0.73	0	0.02	0
chloroplasts	0	0	0	0.02	0	0.26	0	1.8
photosynthetic_cyanobacteria	0.02	0.96	3.45	0.01	0.03	4.46	0	12.79
oxygenic_photoautotrophy	0.02	0.96	3.45	0.01	0.03	4.46	0	12.79
photoautotrophy	0.02	0.96	3.45	0.01	0.03	4.46	0	12.79
photoheterotrophy	0.31	3.83	0	0.49	1.25	0.32	5.04	2.8
phototrophy	0.33	4.79	3.45	0.5	1.29	4.78	5.05	15.59
ureolysis	0	0	0.09	0	0	0	0.01	0
chemoheterotrophy	3.06	7.64	82.42	1.21	5.07	77.65	5.66	26.48

* only processes with share > 0.01 % are shown
